# Electrophoretically prepared hybrid materials for biopolymer hydrogel and layered ceramic nanoparticles

**DOI:** 10.1186/s40824-016-0048-4

**Published:** 2016-02-10

**Authors:** Gyeong-Hyeon Gwak, Ae-Jin Choi, Yeoung-Seuk Bae, Hyun-Jin Choi, Jae-Min Oh

**Affiliations:** Department of Chemistry and Medical Chemistry, College of Science and Technology, Yonsei University, #326, Changjo-hall, Wonju Campus, Yonseidaegil 1, Heungeop-myeon, Wonju, Gangwondo 26493 Republic of Korea; Postharvest Research Team, National Institute of Horticultural and Herbal Science (NIHHS) of RDA, Wanju, Jeollabukdo 55365 Republic of Korea

**Keywords:** Biopolymer, Agarose, Gelatin, Ceramic, Layered double hydroxide, Electrophoretic synthesis, Controlled release

## Abstract

**Background:**

In order to obtain biomaterials with controllable physicochemical properties, hybrid biomaterials composed of biocompatible biopolymers and ceramic nanoparticles have attracted interests. In this study, we prepared biopolymer/ceramic hybrids consisting of various natural biopolymers and layered double hydroxide (LDH) ceramic nanoparticles via an electrophoretic method. We studied the structures and controlled-release properties of these materials.

**Results and discussion:**

X-ray diffraction (XRD) patterns and X-ray absorption spectra (XAS) showed that LDH nanoparticles were formed in a biopolymer hydrogel through electrophoretic reaction. Scanning electron microscopic (SEM) images showed that the ceramic nanoparticles were homogeneously distributed throughout the hydrogel matrix. An antioxidant agent (i.e., ferulic acid) was loaded onto agarose/LDH and gelatin/LDH hybrids, and the time-dependent release of ferulic acid was investigated via high-performance liquid chromatography (HPLC) for kinetic model fitting.

**Conclusions:**

Biopolymer/LDH hybrid materials that were prepared by electrophoretic method created a homogeneous composite of two components and possessed controllable drug release properties according to the type of biopolymer.

## Background

Biomaterials, in a broad sense of the definition, are materials that can be applied to biological systems. They include materials used in medical devices, artificial tissues/organs, bone cement, dental implants, biosensors, catheters, drug delivery systems, hygiene items, etc. [1–3]. In terms of their material properties, biomaterials can be classified as polymers, metals, ceramics, and hybrid materials. Among them, polymers (e.g., poly(lactic acid) (PLA), poly(glycolic acid) (PGA), and poly(lactic-co-glycolic acid) (PLGA)) have been widely studied for soft tissue applications to recover the structure and function of organs. Polymers are desirable because they possess mechanical flexibility, biodegradability, cellular interaction, easy modification, etc. [4, 5]. Natural polymers, like collagen, have been investigated for use as tissue engineering scaffolds [6]. Ceramics are often utilized in hard tissue applications because they have high mechanical strength and chemical stability. For instance, calcium phosphate and hydroxyapatites have been extensively studied for use in mesenchymal stem cell differentiation, bone engineering, and dental implants [7–9]. Metallic biomaterials, such as stainless steel, Ti alloys, and Co-Cr alloys, can be applied in surgical implants or bone tissue engineering applications due to their easy sterilization, high mechanical strength, fracture resistance, and widely available fabrication techniques [10, 11].

Recently, hybrid biomaterials consisting of two or more components have been developed in order to achieve synergic effects. For instance, polymer/polymer hybrids of gelatin, alginate, hyaluronate, and chitosan were reported as wound dressings; these materials have controlled porosity and water uptake properties [12]. An agarose/chitosan hybrid that was suggested by Z. Cao *et al.* achieved reasonable mechanical strength and effective neuronal growth in 3D space [13]. C. Du *et al.* reported a hydroxyapatite-incorporated collagen-ceramic/polymer hybrid that was both bioactive and biodegradable [14]. Y. Ito *et al.* developed hybrids that were composed of biocompatible and biodegradable poly(3-hydroxybutyrate-co-3-hydroxyvalerate) (PHBV) nanofibers and hydroxyapatite in order to achieve high specific surface area, surface hydrophilicity, and enzyme invasion [15].

**Scheme 1:**
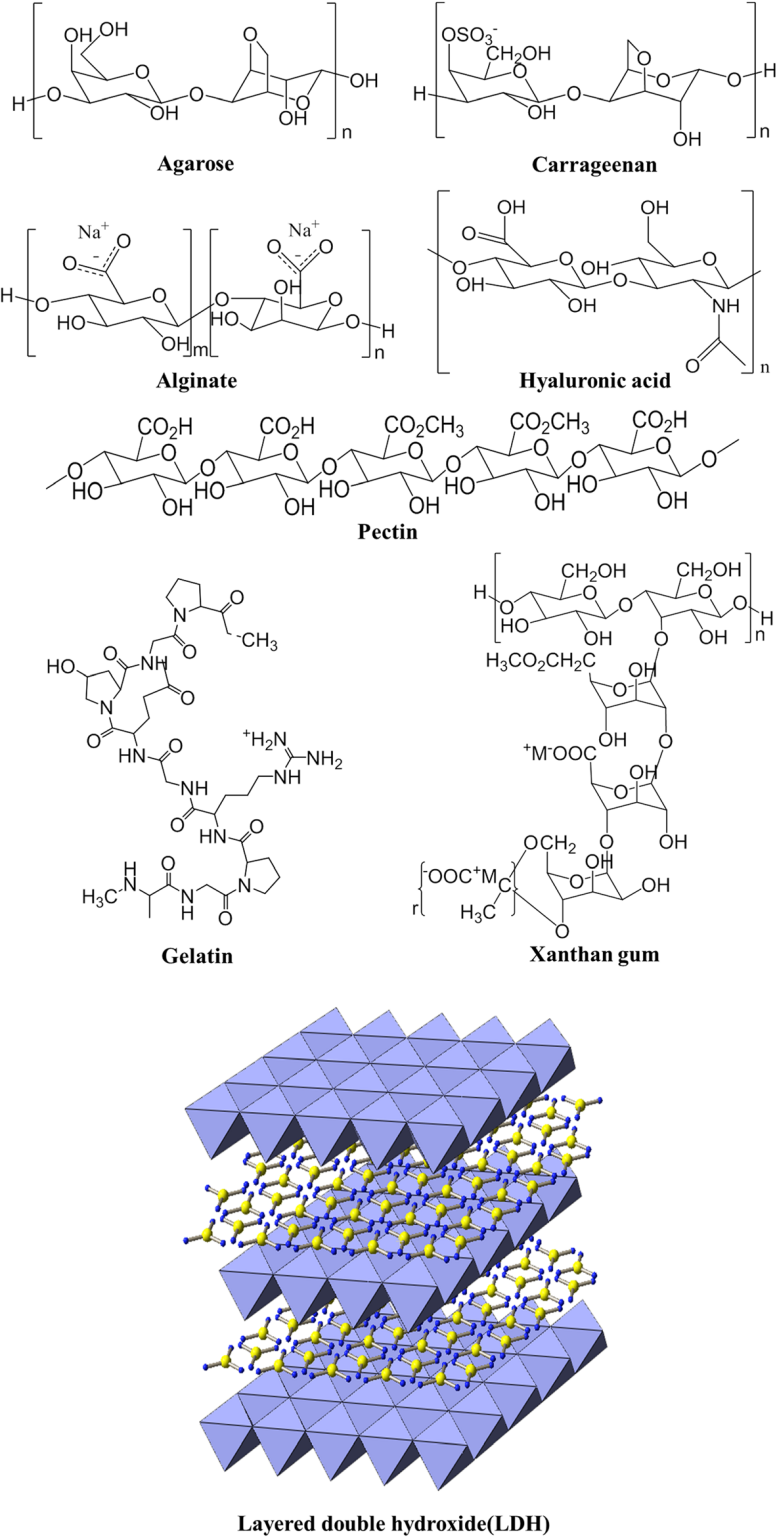
Schematic molecular structures of biopolymers and LDH nanoparticles

In this context, we attempted to prepare polymer/ceramic hybrids consisting of biopolymer hydrogels and layered ceramic nanomaterials. For the preparation of these materials, we utilized electrophoretic hybridization, in which ceramic particles were grown *in situ* in a hydrogel matrix, thereby resulting in the uniform distribution of ceramic nanomaterials [16]. As shown in Scheme 1, several natural biopolymers (that are known to form hydrogels) and ceramic nanomaterials (layered double hydroxide (LDH)) were chosen as the candidate materials. Agarose is a biopolymer of polysaccharide chains that can be utilized as the scaffold in tissue engineering [17, 18]. Gelatin is a mixture of peptides and proteins obtained from the partial hydrolysis of natural animal collagen; this is commercially utilized as a biodegradable polymer [6]. Carrageenan is a polysaccharide polymer extracted from red seaweed and is utilized as a gelation agent or drug stabilizer [19]. Xanthan gum is obtained via the fermentation of carbohydrates with *Xanthomonas Campestris* and is often utilized to control the viscosity of food [20]. Alginate is a characteristic polysaccharide that forms a hydrogel in the presence of divalent metal ions and can be utilized as both wound dressing and scaffolds [21]. Pectins are extracted from the peels of citrus fruits and are widely used as food stabilizers [22]. Hyaluronic acid is used in implant coatings or during ocular surgery due to its high water content in its hydrogel form [23]. The aforementioned biopolymers are known to show a temperature-dependent gel-sol transition and high biocompatibility; thus, they are excellent candidates for use in drug delivery systems and tissue engineering. The ceramic material in this study is layered double hydroxide (LDH), which is composed of positively charged nanosheets and interlayer anions [24]. Due to its biocompatibility and high anionic exchange capacity, LDH has been suggested as a targeted cellular drug delivery carrier or for use in sustained drug release systems.

Herein, we demonstrate possible biopolymer/LDH hybrids made via an electrophoretic method. The obtained hybrids were analyzed in terms of their structure and nanoparticle distribution utilizing X-ray diffraction, X-ray absorption spectroscopy, and electron microscopy. We also investigated the potential of these prepared hybrids in sustained drug release system utilizing an antioxidant agent, ferulic acid, as the model drug.

## Method

### Materials

Agarose (MW: 120 kDa) was purchased from Bio Basic Inc., Canada. Gelatin (from porcine skin), zinc nitrate hexahydrate (Zn(NO_3_)_2_ · 6H_2_O), aluminum nitrate nonahydrate (Al(NO_3_)_3_ · 9H_2_O), sodium bicarbonate (NaHCO_3_), tris(hydroxymethyl)aminomethane (Tris: NH_2_C(CH_2_OH)_3_), and the antioxidant agent (ferulic acid (C_10_H_10_O_4_)) were purchased from Sigma-Aldrich Co. LLC, USA. Ammonia water (NH_4_OH), sodium hydroxide (NaOH), and hydrochloric acid (HCl) were purchased from Daejung Chemicals & Metals Co. LTD., Korea.

### Electrophoresis method

In order to prepare biopolymer/LDH hybrid materials electrophoretically, a home-made electrophoretic kit was utilized. First, the biopolymer powder (1 wt/v% for agarose and 2 wt/v% for gelatin and the other biopolymers) was dissolved in tris-HCl buffer (pH 7.4) at 120 ^o^C. Then, the solution was poured into the center of the electrophoretic kit walled by plastic plates and cooled down to room temperature for 4 h to obtain a cuboidal hydrogel. The cationic metal solution (0.16 M Zn^2+^ and 0.08 M Al^3+^) and the anionic solution (0.08 M NaHCO_3_ and 1 mL NH_4_OH), which were precursors for LDH, were located at each side of the cuboidal hydrogel. Then, electrophoresis was operated with 25 V for 30 min. After reaction, the hydrogel was washed with deionized water and thoroughly dehydrated.

As a reference sample for LDH, ZnAl-CO_3_-LDH (Zn_2_Al(OH)_6_(CO_3_)_0.5_) was prepared by conventional coprecipitation method, as reported elsewhere [24]. Typically, the cationic metal solution (0.063 M Zn^2+^ and 0.0315 M Al^3+^) was titrated with basic solution (NaOH and NaHCO_3_) to pH ~8.5 with vigorous stirring. After 24 h, white precipitates formed. These were centrifuged, washed by deionized water, and then dried.

### Characterization

Prepared hybrids were characterized by X-ray diffraction (XRD), X-ray absorption spectroscopy (XAS), and field emission scanning electron microscope (FE-SEM). In order to identify the crystal structure of the ceramic particles in the hybrid, XRD patterns and XAS spectra were obtained by Bruker D2 phaser with Ni-filtered Cu-Kα radiation (λ = 1.5406 Å) and at the 7D XAFS beam line at the Pohang Accelerator Laboratory (Pohang, Korea), respectively. FE-SEM images, obtained with Hitachi SU-70 at the Korea Basic Science Institute (Gangneung Center, Korea), showed the shape and size of LDH nanoparticles in the hybrids.

### Sustained release test

Ferulic acid (FA), which was the model molecule used in our drug delivery system, was dissolved in deionized water and the pH was adjusted to 7 ~ 8 for clear dissolution. The lyophilized biopolymer/LDH hybrid (approximately 0.5 g) was soaked in 50 mL of the FA solution (0.05 M) for 1 day and then dehydrated. The FA-loaded hybrid was put into 30 mL of saline and supernatant aliquots were obtained at each time point (0, 5, 10, 20, 30, 45, 60, and 120 min) with shaking. After filtration, aliquots were quantified by high-performance liquid chromatography (HPLC: YL9100 YL instrument Co., Ltd.) with C18 column at 35 °C. A mixed solution of phosphoric acid and acetonitrile (ratio = 7:3) was used as an eluent. Aliquots were subjected to HPLC analysis at a flow rate of 1.0 mL/min at 254 and 320 nm with a dual-wavelength UV-visible detector. The percentages of the released amounts of FA in the biopolymer/LDH hybrid were obtained and the kinetics were analyzed with the Elovich model (eq. 1).

1 $$ \mathrm{Q}\mathrm{t} = \frac{1}{\beta } \ln \left(\alpha \beta \right) + \frac{1}{\beta } \ln (t) $$

(Qt: release amount at time t, t: time, α and β: Elovich constants representing the initial release rate and the overall release rate, respectively.)

## Results and discussion

We chose LDH (Zn_2_Al(OH)_6_(CO_3_)_0.5_ (Scheme 1)) as the ceramic nanoparticle because it is known to a biocompatible inorganic material that can effectively stabilize and control the release kinetics of biologically-functionalized anions [25, 26]. As candidates for the hydrogel components, we chose several biopolymers that are commonly utilized as biomaterials and form hydrogels via thermoreversibility (Scheme 1). First, we attempted to make hydrogels with 0.5–3.0 % (wt/v) of the selected biopolymers. This concentration was selected because our preliminary study showed that hydrogels in this range effectively adopt precursors to make ceramic nanoparticles inside hydrogels. As shown in Table 1, most of the biopolymers formed gels within this concentration range, with the exception of hyaluronic acid. Next, we checked whether or not the hydrogels could be manipulated as cuboidal lumps (4 cm × 3.5 cm × 1 cm) in order to apply them to the electrophoretic kit. Unfortunately, neither alginate nor pectin formed cuboids; instead, they made small and separated gel particles. Thus, we carried out electrophoretic reactions on agarose, gelatin, carrageenan, and xanthan gum. After 30 min of reaction, we checked for the formation of ceramic nanoparticles by looking for a color change in the hydrogels; the translucent gels turn opaque upon the formation of nanoparticles. While agarose and gelatin clearly showed color changes, carrageenan and xanthan gum gels did not exhibit opacity. Compared to agarose and gelatin, lots of anionic sites in the carrageenan and xanthan gum can be directly coordinated with metal cation-forming complexes, which can hinder the formation of ceramic nanoparticles.

**Table 1:** Hydrogel candidates for the electrophoretic preparation method

Biopolymer	Hydrogel formation	Cuboidal hydrogel formation	Electrophoretic hybrid formation
Agarose	**○**	**○**	**○**
Gelatin	**○**	**○**	**○**
Carrageenan	**○**	**○**	×
Xanthan gum	**○**	**○**	×
Alginate	**○** (with divalent cations)	×	×
Pectin	**○** (with divalent cations)	×	×
Hyaluronic acid	×	×	×

**Fig. 1:**
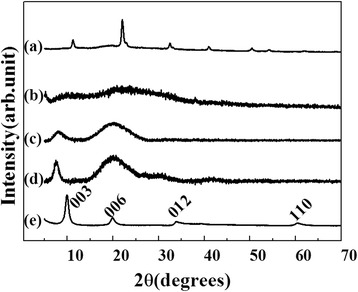
XRD patterns of (**a**) agarose film, (**b**) agarose/LDH, **(c)** gelatin, (**d**) gelatin/LDH, and (**e**) coprecipitated ZnAl-LDH nanoparticles

In order to confirm that the particles inside of the hydrogels had a LDH structure, we further characterized the agarose/LDH and gelatin/LDH hybrids with XRD and XAS. Figure 1 shows XRD patterns of the biopolymer hydrogels, hybrids, and conventionally prepared LDH. Dried agarose showed crystalline peaks at 11.15, 22.06, 32.45, 40.90, and 50.48°; these were attributed to the ordered stacking of polysaccharide rings [27]. However, the agarose/LDH hybrid showed an amorphous pattern that had neither agarose nor LDH peaks. The formation of ceramic LDH nanoparticles inside of the hydrogel perturbed the ordered stacking of polysaccharide, thereby reducing the crystallinity of the hydrogel. The LDH nanoparticles that formed inside of the hydrogel might be too small to be detected in their crystalline form. The dried gelatin showed broad XRD peaks around 8.00 and 20.10°, which correspond to the α-helix and triple helical structures [28]. Gelatin/LDH showed an XRD pattern that was similar to gelatin, but the degree of structure perturbation of LDH was less serious in gelatin than in agarose.

**Fig. 2:**
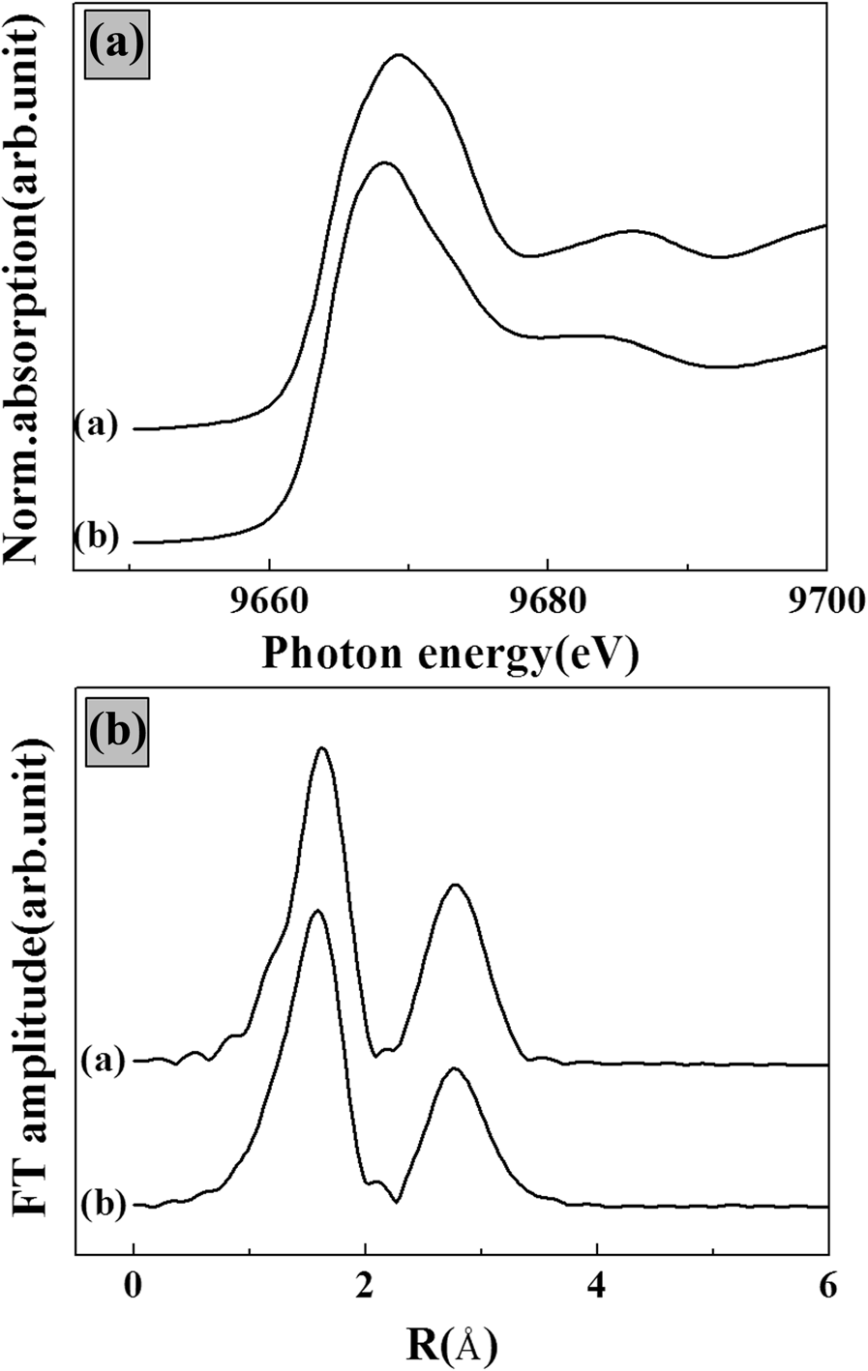
**a** Zn K-edge XANES spectra and **b** Fourier-transformed EXAFS spectra for (*a*) coprecipitated ZnAl-LDH nanoparticles and (*b*) agarose/LDH

Because we could not clearly confirm that the ceramic particles forming inside of the hydrogel were LDH-phased particles, we carried out XAS analysis. XAS shows the local structures around certain metal ions, enabling us to determine whether the structure of the particles matched that of LDH. XAS spectra can be divided into two regions: the X-ray absorption near edge structure (XANES) and the extended X-ray absorption fine structure (EXAFS). XANES provides information about metal ions, such as the oxidation number and local symmetry, while EXAFS shows the bond length between the target metal and nearby atoms as well as disorder in coordination bonds. As shown in Fig. 2a, both the hybrid and ZnAl-LDH showed the main edge of the Zn K-edge at 9664.08 eV; this suggests the existence of the Zn ion 2+ oxidation state [29]. Because neither XANES spectra showed a pre-edge, Zn(II) in both samples was determined to have fully-occupied 3d electrons and to possess octahedral symmetry [30, 31]. Generally, Zn(II) in biological circumstances tends to possess tetrahedral symmetry, while the LDH phase contains Zn(II) at its octahedral sites. Thus, we can infer that the hybrid contained LDH nanoparticles. Although the XANES spectra of ZnAl-LDH and the hybrid showed similar patterns, we found that the secondary peak (around 9685 eV) and the oscillation pattern beyond 9680 eV were slightly different according to the sample (Fig. 2a). This subtle difference was further evaluated with Fourier transformed-EXAFS spectra. As shown in Fig. 2b, spectra of both samples showed first-shell peak at R = 1.63 Å (non-phase-shift-corrected) and second-shell peak at R = 2.76 Å (non-phase-shift-corrected). These had similar shapes, which suggested that the inorganic particles inside of the hydrogel have the same crystal structure as ZnAl-LDH. In order to quantitatively evaluate the structure, including features like the bond lengths and structural disorder caused by thermal vibration, we carried out FEFF6-code analyses. The first- and second-shell peaks were attributed to Zn-O and Zn-(Zn or Al), respectively. The calculated bond lengths of Zn-O were 2.09 Å and 2.06 Å for conventional ZnAl-LDH and hybrid LDH, respectively. Those of Zn-(Zn or Al) were 3.15 Å and 3.19 Å, respectively. The Debye-Waller factors, which reflect the structural disorder, were 0.007 (first-shell) and 0.017 (second-shell) for conventional LDH and 0.010 (firs- shell) and 0.029 (second-shell) for the nanoparticles in the hybrid. The slightly different bond lengths and increase in the Debye-Waller factor for the hybrids suggested that the LDH structure inside of the hybrid was slightly distorted. This difference is thought to originate from the small particle size of LDH inside of the hydrogel as compared to those of the conventionally prepared ZnAl-LDH.

**Fig. 3:**
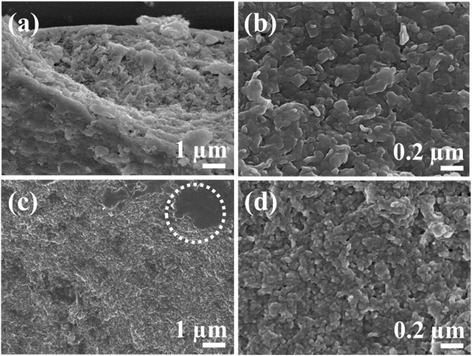
FE-SEM images of (**a**), (**b**) coprecipitated ZnAl-LDH and (**c**), (**d**) agarose/LDH

In order to investigate the size and morphology of agarose/LDH, as compared to conventionally prepared ZnAl-LDH particles, we carried out SEM measurements (Fig. 3). Although conventionally prepared LDHs do not clearly show primary particles due to agglomeration (Fig. 3(a)), we can determine that the primary particle size is ~20 nm in the magnified images (Fig. 3(b)). SEM images of agarose/LDH showed both a polymeric phase with a smooth surface (dotted circle in Fig. 3(c)) [8] and a widely distributed particulate phase. We magnified the particulate phase to observe the small size (20–30 nm) and spherical shape of the LDH nanoparticles inside of the hydrogel. The significantly smaller size of LDH particles in agarose/LDH, as compared to conventional ZnAl-LDH, might be attributed to the restricted crystal growth of LDH particles in the hydrogel polymer network.

**Fig. 4:**
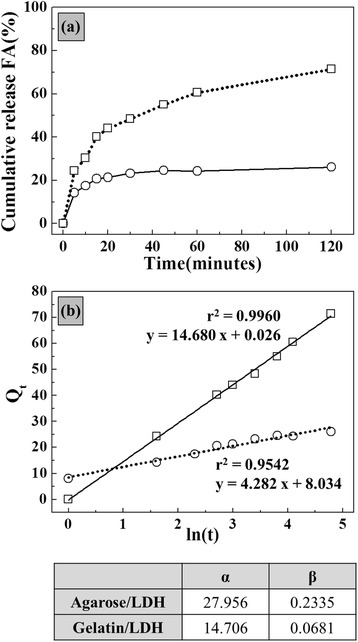
**a** Time-dependent ferulic acid release patterns from agarose/LDH (open circle) and gelatin/LDH (open square) and **b** their kinetic model fitting results and kinetic constants

Through various characterizations, we confirmed that a biopolymer/LDH hybrid with small and uniform ceramic LDH nanoparticles can be prepared by an electrophoretic method. Because both components of the hybrid (i.e., the biopolymer and LDH) are potential biomaterials, the resultant hybrid can be utilized in bio-medical applications [25, 32]. Taking into account the fact that LDHs are attractive materials for sustained release drug delivery [25, 26], we attempted to investigate the drug release patterns of agarose/LDH and gelatin/LDH hybrids. We chose an antioxidant agent (i.e., ferulic acid (FA)) because it has been reported that FA can be incorporated into LDH for drug delivery [33, 34]. In the experiments, lyophilized hybrids were soaked in an FA solution and again lyophilized. The samples showed FA contents of 49.93 wt % and 13.63wt % for the agarose/LDH and gelatin/LDH hybrids, respectively. Figure 4a shows the time-dependent and cumulative FA release curve for each hybrid, demonstrating releases of 26.09 % and 71.41 % after 2 h for agarose/LDH and gelatin/LDH, respectively. We fitted the time-dependent curves to well-known release kinetic curves, such as the first order Elovich equation, parabolic diffusion, and power function (data not shown). It was determined that only the Elovich model showed a coefficient of determinant (r^2^) higher than 0.9. The Elovich model hypothesizes heterogeneous diffusion via the adsorption-desorption of ions and is often utilized to study the release kinetics of clay materials or LDHs [35, 36]. Two constants (α and β) are important in Elovich model analysis; these are related to the initial release rate and the desorption kinetics, respectively [35]. It has been reported that sustained release systems exhibit small α and large β values [16]. The α values of agarose/LDH and gelatin/LDH were determined to be 27.956 and 14.706, showing higher initial desorption of FA from agarose/LDH than from gelatin/LDH. The β values were 0.2335 and 0.0681 for agarose/LDH and gelatin/LDH, respectively, suggesting that agarose/LDH possesses superior sustained release properties. The different FA release kinetics of the two hybrids might be related to the polymer structure and polymer-LDH interactions inside of the hybrids. Agarose, which consists of polysaccharides with δ^−^ sites, showed higher interaction with positive LDH nanoparticles compared to gelatin, which consists of polyaminoacids. The uniform distribution of LDH nanoparticles inside of the agarose matrix and the effective interaction between agarose and LDH can effectively block pathways of the FA molecules, reducing the total release amount and the release rate.

## Conclusions

We prepared polymer/ceramic hybrid biomaterials via an electrophoretic preparation method. These materials are suitable for the homogeneous formation of ceramic nanoparticles in a hydrogel. We chose agarose and gelatin as the polymer component and LDH nanoparticles as the ceramic component. All of these materials have adequate biocompatibility and are widely applied in biomedical fields. We identified the crystal structure of LDH nanoparticles by XRD and XAS, which showed that the ceramic nanoparticles inside of the hybrid had the desired LDH structure. By analyzing SEM images, LDH nanoparticles were determined to be homogeneously formed in the hydrogel as we expected. Ferulic acid (the drug model molecule used in our study) was well-loaded onto the biopolymer/LDH hybrid and was released in a sustained manner.
